# Transcriptomic data sets for *Synechococcus elongatus* PCC7942 transformant cells expressing mycosporine-2-glycine under salt stress conditions

**DOI:** 10.1128/mra.01342-25

**Published:** 2026-02-12

**Authors:** Tomoki Tsuboi, Rungaroon Waditee-Sirisattha, Hakuto Kageyama

**Affiliations:** 1 Graduate School of Environmental and Human Sciences, Meijo University 12942, Nagoya, Japan; 2 Department of Microbiology, Faculty of Science, Chulalongkorn University 26683, Bangkok, Thailand; 3 Department of Chemistry, Faculty of Science and Technology, Meijo University 12942, Nagoya, Japan; Indiana University, Bloomington, Bloomington, Indiana, USA

**Keywords:** cyanobacteria, mycosporine-like amino acid

## Abstract

RNA sequencing was performed on *Synechococcus elongatus* PCC 7942 transformant cells expressing mycosporine-2-glycine under salt stress. The data provide global transcriptomic profiles of cyanobacteria when the mycosporine-like amino acid biosynthetic pathway is heterologously expressed.

## ANNOUNCEMENT

Mycosporine-like amino acids (MAAs) are small, water-soluble compounds that absorb ultraviolet (UV) radiation and are widely distributed among various organisms, including red algae, sea stars, corals, dinoflagellates, and cyanobacteria (1). These compounds are thought to play protective roles against environmental stresses, such as UV exposure and salinity (2). The cyanobacterium *Synechococcus elongatus* PCC 7942, a widely used model organism, does not naturally produce MAAs (3). We previously generated a mycosporine-2-glycine (M2G)-producing *Synechococcus* strain by introducing the *mysABCD* gene cluster derived from the halotolerant cyanobacterium *Halothece* sp. PCC 7418 (4). This M2G-expressing strain accumulates M2G in response to salt stress (4). Here, we report RNA-seq analyses of the M2G-expressing strain and a control strain under 0.3 M NaCl to describe global transcriptional responses associated with M2G production under salt stress.

The M2G-expressing strain was generated by introducing the *Halothece mysABCD* genes into *Synechococcus* using plasmid pUC303. *mysA* (PCC7418_1590) and the *mysB–mysD* operon (PCC7418_1078–1076) were cloned into the BamHI and XhoI sites, respectively. For each insert, approximately 500 bp upstream of the coding sequence was included to retain the native promoter region. Transformation of *Synechococcus* with the vector was performed by natural transformation, and transformants were selected on streptomycin (25 µg/mL). The control cells harbored the empty plasmid pUC303.

Both strains were grown in BG-11 medium supplemented with 25 µg/mL streptomycin at 30°C under continuous illumination for photoautotrophic growth (40 µmol photons m⁻² s⁻¹). Cultures were initiated in three independent flasks from separate starter cultures. When cultures reached an OD₇₃₀ of 0.3–0.4, cells were harvested by centrifugation at 2,290 × *g* for 15 min at 4°C as Day 0 samples. Three independent 15 mL cultures were pooled to generate one composite sample per condition. For salt stress treatment, cells were harvested from three independent 70 mL cultures by centrifugation at 2,290 × *g* for 15 min at 30°C, resuspended in fresh BG-11 medium supplemented with 0.3 M NaCl, and cultivated for 72 h. Cells were then harvested by centrifugation as described above to obtain Day 3 samples, and the three cultures were pooled at equal volumes to generate one composite sample per condition.

Total RNA was extracted using the RNeasy Plant Mini Kit (Qiagen) on a QIAcube Connect System after cell disruption in Buffer RLT (containing 2-mercaptoethanol) using a multi-beads shocker (Yasui Kikai) at 1,500 rpm for 2 min. RNA quantity and quality were assessed using a Quantus fluorometer (Promega), QuantiFluor RNA System, and Fragment Analyzer System (Agilent Technologies). Ribosomal RNA was removed using the Ribo-off rRNA Depletion Kit V2 (Bacteria; Vazyme). RNA libraries were prepared with the MGIEasy Fast RNA Library Prep Set (MGI Tech) quantified using a Qubit 3.0 fluorometer and dsDNA HS Assay Kit (Thermo Fisher Scientific) and assessed for quality with an Agilent 2100 bioanalyzer. Circular DNA and DNA nanoballs (DNBs) were prepared using the MGIEasy Dual Barcode Circularization Kit and the DNB Rapid Make Reagent Kit (MGI Tech), respectively, and loaded onto the DNBSEQ-T7 platform (MGI Tech) for paired-end 150 bp sequencing.

Adapters and low-quality reads were removed using cutadapt (v4.0) (5) and sickle (v1.33) (6). Clean reads were then mapped to the *S. elongatus* PCC 7942 reference genome (GenBank: GCF_000012525.1) using Bowtie2 (v2.5.0) (7). SAM files were converted to BAM format, sorted, and indexed using samtools (v1.17) (8). Gene-level read counts were obtained using featureCounts (v2.0.3) (9) and normalized to transcripts per million. All software was run with default parameters. A summary of reads and mapping results is present in Table 1.

**TABLE 1:** Data set descriptors and run accessions

Sample	Strain	Time point	NaCl	Total filtered paired reads	% of reads mapped	Run accession
Control-D0	Control (empty plasmid)	Day 0	0 M	23,713,430	87.98	DRR795492
Control-D3	Control (empty plasmid)	Day 3	0.3 M	24,726,675	92.86	DRR795493
M2G-D0	M2G-expressing strain	Day 0	0 M	24,867,170	96.38	DRR795494
M2G-D3	M2G-expressing strain	Day 3	0.3 M	24,958,555	93.25	DRR795495

**Fig 1:**
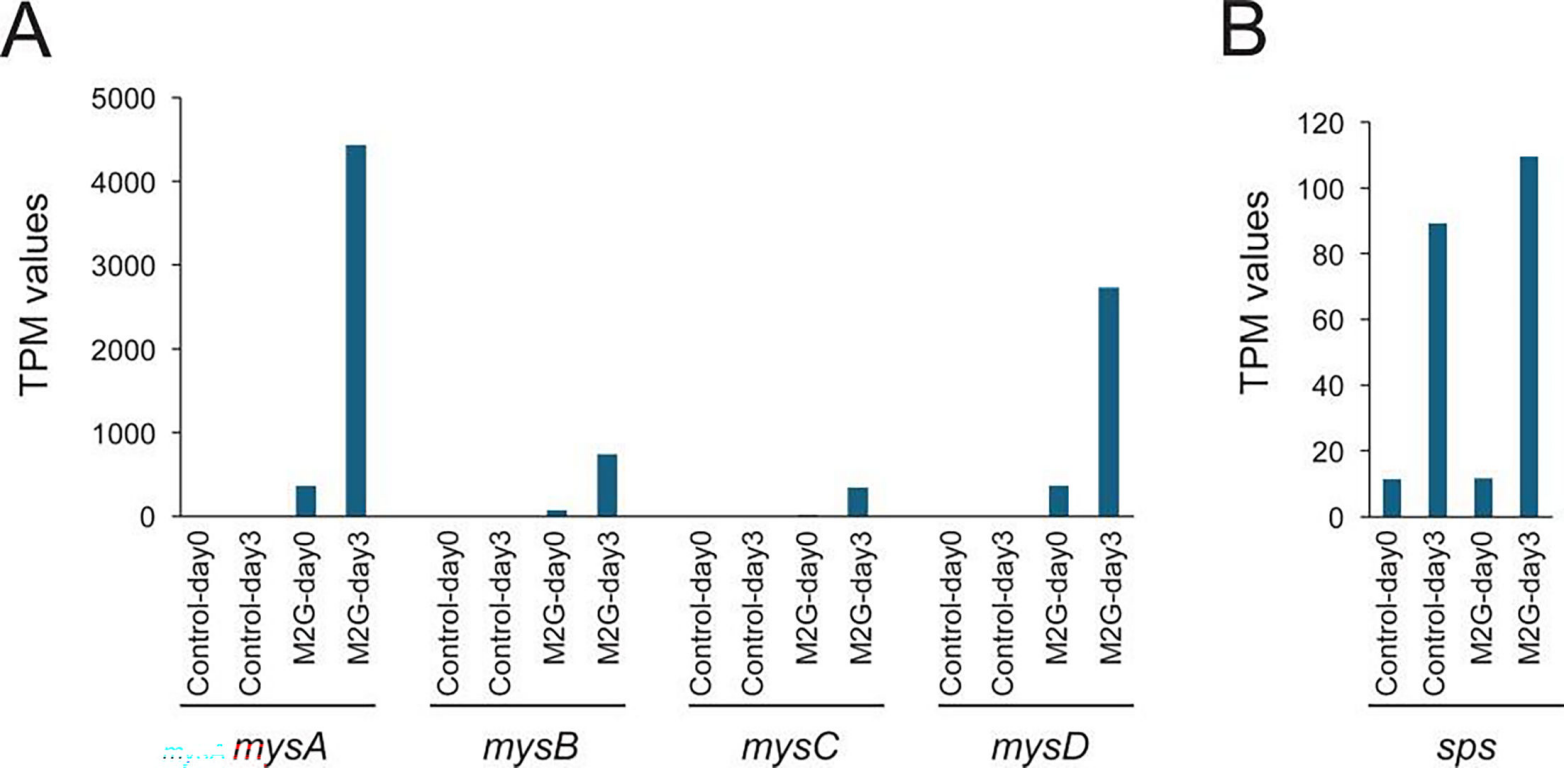
Transcripts per million (TPM)-based expression examples supporting data set utility. (**A**) TPM values for transcript mapping to the *mysABCD* genes (PCC7418_1590, 1078, 1077, and 1076) in the empty-vector control and M2G-expressing strains at days 0 and 3 under 0.3 M NaCl. (**B**) TPM values for sucrose-phosphate synthase (*sps*; Synpcc7942_0808) in the same samples.

Because each condition is represented by a single sequenced library (a pooled composite of three flasks), differential-expression patterns are provided for exploratory purposes and should not be interpreted as statistical evidence of differential expression. As an example of data set utility, transcripts mapping to the introduced *mysABCD* genes were detected in M2G-expressing strain and were higher under salt stress while remaining undetectable/near-background in control strain (Fig. 1A). In addition, expression of sucrose-phosphate synthase (Synpcc7942_0808) increased under salt stress in both strains (Fig. 1B), consistent with a known salt-stress response in *Synechococcus*, where sucrose accumulates as a compatible solute (10).

## Data Availability

The raw sequencing data generated in this study are publicly available in the DDBJ Sequence Read Archive under accession DRA023690 (BioProject PRJDB38059). The four runs correspond to the conditions in Table 1 (DRR795492, DRR795493, DRR795494, and DRR795495).
